# Analysis of Medical Evidence, Comprising Directions for Practitioners, as Witnesses in Courts of Justice

**Published:** 1825-07-01

**Authors:** 


*An Analysis of Medical Evidence:—comprising Directions for Practitioners, in the View of becoming Witnesses in the Courts of Justice; and an Appendix of Professional Testimony.* By John Gordon Smith, M.D. 8vo. pp. 386.

This, Dr. Smith informs us, is the first of several distinct works on particular subjects of medical jurisprudence, which he has projected, and which, we sincerely hope, he will accomplish in due time. Of elementary or general works on this branch of science, he thinks we have now enough—and that, therefore, the labours of those who are still inclined to work in this department will be most advantageously exerted "by concentrating their powers upon individual subjects." Under this impression Dr. Smith has selected the topic of *medical evidence*, one of the most urgent and important subjects, he conceives, within the whole range—a subject, the elucidation of which has been hitherto almost unattempted, with the exception of a few detached observations in the systematic works of Dr. Paris and Dr. Smith himself.

"The work is composed of two distinct parts: that which is more strictly original, and that which may, at first sight, appear to be little else than long and multifarious extracts, from sources of easy access. I might, perhaps, justly point to this portion of the volume as being the most important; while, in all probability, not a few may look upon it as the most interesting. I have to trouble the reader with a few prefatory remarks, with respect to both." xii.

In the first part, it has been Dr. Smith's object to excite the profession to think for themselves. The basis of this portion of the work was delivered, *ex ore*, before the medical society of London, and was there well received. We anticipate a similar reception from the public at large to this more developed performance.

Our author opens his work with an expression of surprise that no provision is made in this country for the tuition of medical ethics—a branch which he thinks is intimately connected with *medical jurisprudence*, for which also there is no provision in the schools of this country—England. This neglect, he observes, has brought down much unjust reproach upon medicine, as he fears not to assert that "the instances in which medical witnesses have come down from any judicial examination of importance, without suffering more or less injury, have not been many"—that those in which credit has been gained, have been fewer still—and that no instance is on record where "an individual reputation has been thereby *established*." We confess that this last circumstance excites in our mind neither surprise nor regret. It would not, we imagine, be well that a medical reputation should be *established* by the mere *eclat* of a display in a Court of Justice. We believe it is pretty well proved by universal experience, that a professional reputation, in medicine, is not to be acquired either by writings or speeches. These may put a man in the way of having his talents tested a little sooner—but we believe that nothing but the solid acquirement of knowledge at the bedside of sickness will ever *establish* a physician's or a surgeon's reputation. God forbid it should be otherwise! At the same time we honour the man who attempts to improve the appearance of a medical practitioner in the *witness-box*.

CHAP. I. *On Evidence.* "Witnesses in general, and medical witnesses among others, have little direct business, either with the law or the practice of evidence." If he tends to wander from the proper point he will be promptly checked. At the same time, our author observes, that it cannot be idle on the part of the medical practitioner, to acquire some knowledge of the manner in which he is to perform the important office of delivering evidence. It is considered that every man of liberal education should possess some knowledge of his municipal and political relations to the state—as well as of his individual rights and privileges. It is probable, as Dr. Smith observes, that medical men are less acquainted with jurisprudential matters than others of the same rank in society, from the circumstance of their not being called upon to serve on juries. The remainder of this chapter is taken up with the forms, or we might say, the formalities of swearing witnesses, the bearings of which on medical jurisprudence we confess we cannot always clearly see. Thus, we are informed by our author that Jews cover their heads and swear on the Pentateuch—those of the Roman and reformed Episcopal persuasions lay the right hand upon the Bible—Quakers merely make a solemn affirmation, &c.

II.—The second chapter is on the obligation to give medical evidence. It is almost unnecessary to say that a medical practitioner is liable to be called on for evidence, in common with his fellow citizens; but he is also liable to be subpœnaed in his *professional character*, to the great inconvenience of his daily avocations. But these attendances, as Dr. Percival remarks, in his Medical Ethics, are "appropriate debts to the community, which neither equity nor patriotism will allow to be cancelled." Here Dr. Smith has enveloped his ideas in such endless and tortuous circumlocutions, that we find it extremely difficult to unravel his real meaning or object. Thus, having given the attorneys a little touch on their tenacity of remuneration for judicial services, Dr. S. goes on as follows:—

"But it will be said that this retort is futile, because *we* are bound, in a way that others are not, to do all the labour that can possibly be required of us—even to build churches and bridges, cut canals, and make roads; supply the country with jails, attend them as turnkeys, and finally, 'to aid *the execution* of public justice,' by performing, without reluctance, the office that may be required from us, by a public functionary who shall be nameless, as soon as, in the rapid course of modern improvement, we shall become 'qualified by professional knowledge,' so to do." 30.

We put it to Dr. Smith's good sense, of which he has no lack, whether such passages as the above do not too closely border on the caricature, and consequently defeat the object which the author may have in view. We are ready to grant that attendances on Coroner's Inquests and other judicial proceedings, without any adequate remuneration, are very inconvenient, not to say oppressive, on the medical practitioner; but of what use is it to fill whole pages of a medical work with complaints on the subject. There is no redress. And if there was any prospect of redress, the appeal to those aggrieved can do no good, but only increase our discontent.

III. *Perplexities of Medical Witnesses.* The liberty which the advocate claims is often applied to the unworthy and unmanly purpose of unhinging the witness rather than eliciting truth. This, perhaps arises, in a great measure, from the frequent occasion which lawyers have of sifting and exposing characters of a depraved cast, so commonly met with in courts of justice. They are necessarily accustomed to mistrust, and estimate very low the claims of those they generally meet at the criminal bar. "Upon such occasions a timid or a stupid (medical) man may be ruined with his *eyes open*, because he is not so adroit at *opening his mouth*."* Doubtless a medical man, of the very first acquirements in his profession, may be thrown off his guard upon such occasions, and thus cut a ridiculous figure. We do not see that there is any thing to help him but his own shrewdness and circumspection—in short, it behoves him, as the common saying is—"to have his wits about him."

"Caution in speaking, as well as in preparing to speak, should be observed by him; and he should on no account answer a question till he clearly understands it, or pretend to frame an answer when he knows nothing of the matter. He should avoid conjectural observations, and continually bear in mind that what he says must stand upon record as signifying what he means. It will not do, under such circumstances, to receive one statement first, and afterwards substitute another. Such a practice would strike at the foundation of evidence. If it were allowable to honest men, it would be claimed by rogues; and justice could never be duly administered." 43.

* It is this kind of point and wit which we find fault with in Dr. Smith's work.

This chapter closes with some animadversions on those who have thrown doubts on the power we possess of ascertaining whether or not a child has breathed, in cases of infanticide. We hope Dr. Smith, in his promised work on this subject, will clear away some of the obscurities and difficulties which have hitherto attended the various tests which have been resorted to for solving the problem.

IV. The fourth chapter is "on the difference of courts"— and is chiefly occupied with a rather tedious disquisition on the importance of medical evidence—and particularly on Coroner's Inquests. The following graphic description of the latter court is in Dr. Smith's peculiar manner and style.

"When we proceed to attend upon a Coroner's jury, we are not perhaps very likely to be reminded of the importance of the occasion by the aspect of the court. Throughout the kingdom (for the usage at large is the proper example to be cited, and not the custom of a particular place, least of all of the metropolis,) a medical practitioner, living in the neighbourhood where the transaction is going on, will in all probability find that the court, or tribunal, or persons to whom he is immediately answerable, are composed of those familiarly known to him; and very likely they may rank a step or two lower in the scale of society than he is habituated to associate with. The place of assembly is probably a mean apartment in a village ale-house, redolent of nicotian; and the beau-ideal of a 'Crowner's quest,' is very much the same as that of a *country-club;* saving that in the latter, the business is possibly discussed with more patience, and *the truth* is more frequently elicited; *in vino veritas—nonne in cervisiâ?* Too often are important grounds for inquiry slurred over: and evidence is frequently taken in such a way as manifestly shews that it is not wanted, any further than may be necessary to make a sufficient shew on the face of the proceedings. This being accomplished, if no troublesome person be present to disturb the even tenour of the way, and no unlucky juryman of sufficient intelligence to confuse the affair by putting an awkward question, the coroner tells the jury what they ought to do; the jury do as they are bid, and every body gets away in good time for dinner, or for supper, as the case may be." 59.

"Not long ago, an inquest was held at a short distance from London, the circumstances leading to which fell under my immediate observation, and were such as to induce me to attend as a spectator. The object was to inquire as to the manner in which a young unknown female came by her death. The body had been found floating in the Thames, and had been several days, apparently, in the water. The occasion was a perfect realization of what I have stated. There were from twelve to twenty honest tradesmen sitting round the public-house parlour; and the parish-beadle in attendance as officer of the court, with two or three witnesses—all grumbling, because Mr. Coroner was a little behind his time. It was the duty of the parish-surgeon to have attended, and he had been duly summoned for the purpose; but, he neither came, nor sent any one in his place, (probably in this he acted conscientiously,) nor forwarded any excuse; nor did the court seem to care in the least about the matter—probably they thought it as well that he neither gave them nor himself any trouble. Another practitioner, however, secured his admission, by a voluntary offer to be of whatever service was in his power. After going through the formalities, from 'O *yes*,' down to 'all persons not of the jury must withdraw,' they returned a verdict of 'Found drowned.' There was no doubt as to the case being one of accidental drowning, even in my own mind; but, as I thought the body should have been opened, and had some knowledge of the foreman, who on his part was aware that such things had formed a prominent object of my studies, I signified to him that their verdict was unwarranted, for that although they had proof enough as to the *finding*, there had not been a rag of evidence to shew that the deceased had been *drowned*. The honest and well-intentioned official went back instantly to his brethren with a view of getting them to re-consider their verdict; but that was a task not to be accomplished by a mere mortal juryman; it was too late—too late in the proceedings, and what was more, too late in the day. It was '*nunc est prandendum*,'—there it ended, and the shadows closed upon a niggard sod that covered—nobody-knew-whom, and few cared what; a suicide, or a victim of complicated violence: the coroner filled up his paper, and the jury went to bed as they had done the night before, *requiescere in pace*; which was more than was supposed to have fallen to the lot of the poor stranger." 61.

V. The fifth chapter is on "party bias;" and contains a long and serious admonition to the medical witness—not to lean to either side—but to tell the truth, the whole truth, and nothing but the truth.

VI. The sixth chapter is entitled—"of the most advisable manner of giving evidence." The opening of the chapter is calculated to damp expectation—if any man could be so insane as to expect *rules* for giving evidence, under "circumstances that vary like the aspect of the clouds, and cannot be fixed as a basis for instruction."

"To this chapter one might fancy all the lame portion of the community hastening, regardless of what precedes or what follows, in the hope that a miraculous healing will attend the dip. But, alas, my friends! this is not a pool for the cure of your malady. If such be your views of your own case, many of you must be too far gone to hope for relief, and if so, all you can rely upon for comfort is the chance (that has happened to some) of being able to avoid exciting causes. There is no nearer road to the realization of your wishes than to begin at the beginning of that knowledge which alone can stand you in stead, and which, as it is not the object of the whole *volume* to impart it, you ought not to expect from any individual chapter." 77.

The four remaining chapters we must entirely pass over, as totally insusceptible of analysis. They will be found well worthy of perusal by all those who wish to prepare themselves for the important office of bearing witness in Courts of Justice.

We now come to the second part of the work, entitled the APPENDIX, and occupying more than 200 pages of closely printed letter-press. It is divided into two parts—the first "consisting of additional notes and elucidations"—the second, "consisting of medical depositions, examinations, &c." This portion of the work is, in reality, worth treble the price affixed to the whole performance, and does great credit to the labour and research of our ingenious author.

We shall be able to glance but superficially over the immense number of topics broached or discussed in this Appendix—still we shall probably not employ a few pages uselessly on this part of the work.

*Medical Ethics.* Under this head our author makes some pertinent remarks on the London schools of medicine. He thinks there is a general attempt, on the part of teachers, to execute *too much*—that is, they attempt too many branches. Secondly, there is not time enough to teach. A course of lectures has no definite meaning. One man must make his course subservient to the convenience of attending that of another, and so on. If he do not, he will get no pupils.

We were grieved to find a man of so liberal a turn of mind as Dr. Smith, give way, *and that under the head of Medical Ethics*, to sarcastic remarks like the following.

"*Thirdly*,—There are too many *professors*. Look for example at the men-midwives, to whom a course of lectures, or a supposed *squad of pupils*, is more essential now-a-days than a *pair of forceps*. They are all *Doctores* too, with very few exceptions; and must have become so, in order to entitle themselves to be called *teachers*, it not being requisite for practice—the College of Physicians having ceased to take cognizance of Obstetrics." 162.

Dr. Smith does not seem to know that, both in the metropolis, and throughout the whole kingdom, there is not a more able, intelligent, and we fearlessly add, *useful* class of practitioners than the *physician-accoucheurs*.

Dr. Smith takes a glance at the Edinburgh school of medicine, and seems to take umbrage at the regulation lately made, compelling physicians to attend a course of *midwifery*—"while they have omitted that *magnificent and far more important branch*"—medical jurisprudence !* Much as we admire the *magnificence* of legal medicine, we cannot allow that it is *far more important* than midwifery. We apprehend that Dr. Smith will ultimately find this sentiment of ours in accordance with that of the public, of the profession—of fathers, mothers, brothers—and even pupils. We will here take the liberty of remarking that, although we would condemn the academical law that should overlook legal medicine, as a branch of study, we highly applaud the clause that renders the study of midwifery imperative. When we consider the numerous, and we might safely say the *peculiar diseases* to which the pregnant and parturient state is subject, we cannot but wonder that the study of midwifery should not have, long ago, been made essential to the qualifications of the physician, who is called in upon all cases of danger. Who would trust his wife or sister, when labouring under a dangerous puerperal disease, in the hands of a physician who considered the study of midwifery totally beneath his notice, and who chose rather to employ a leisure hour in the study of forensic subtleties? Such physician must look for practice at Coroner's inquests, and expect his fees in Westminster Hall.

"*Revelation of Secrets.*—There is a charm about the name of GREGORY, of which I am not afraid to avow the influence,—the less so, as their day is apparently gone by. Although the name be not extinct, at present we miss the light that till of late shone so brilliantly on medicine. Let this be a sufficient apology for the following quotation:—

"'Secrecy is particularly requisite where women are concerned. Independently of the peculiar tenderness with which a woman's character should be treated, there are certain circumstances of health, which, though in no respect connected with her reputation, every woman, from the natural delicacy of her sex, is anxious to conceal; and in some cases, the concealment of these circumstances may be of consequence to her health, to her interest, and to her happiness.' These are the words of the author of 'The Father's Legacy to his Daughters.'" 170.

This is good advice. But we confess we do not see the necessity for occupying nearly a page with the well-known oath of Hippocrates, which, although it contains many good ethical precepts, is, at the same time, lumbered with many items which no man could safely swear to in the present state of so-

* Dr. Smith will be gratified to find that the Senatus Academicus have not omitted legal medicine.

ciety. We do not merely allude to the absurd oath against lithotomy; but to many other injunctions, as swearing to provide for his master, and teach his master's children their profession gratis. These would be cumbrous clauses in a modern indenture! We quote the following passage with approbation.

"*Medicine not a proper Subject of Traffic.*—I do not mean to hint that men should not live by their calling; but, as a liberal profession, I would insist that the healing art should be considered only as the practical application of a science, to which all men of extensive education might do well to devote some attention. As far as the bodily structure *meets the eye*, this is very widely the case; hair, eyes, ears, teeth, nails, skin, and their accidents and derangements, are the objects of considerable solicitude, on the part of many who would in no way busy themselves by attending to corresponding matters, that equally and more vitally interest them. Dr. Gregory argues well for the study of medicine on the part of gentlemen who may not intend to practise it. The closing words of his lectures are these:—

"'I hope I have advanced no opinions in these lectures, that tend to lessen the dignity of a profession which has always been considered as most honourable and important. But I apprehend this dignity is not to be supported by a narrow, selfish, corporation spirit; by self-importance, by a formality in dress and manners, or by an affectation of mystery. The true dignity of physic is to be maintained by the superior learning and abilities of those who profess it, by the liberal manners of gentlemen, and by that openness and candour which disdain all artifice, which invite to a free inquiry, and thus boldly bid defiance to all that illiberal ridicule and abuse, to which medicine has been so much and so long exposed.'—*Gregory.*"

On the subject of "medical liberality," Dr. Smith makes some good observations, and triumphantly proves, that medical men make more sacrifices to humane feelings than men of any other learned profession, not even excepting the Church. We extract the following anecdote:—

"Shall I be pardoned an anecdote? It is *literally* a fact, and very widely applicable. A surgeon of my acquaintance, of most liberal views and conscientious principles, was called to see a person in the garb of a gentleman, who, in travelling through the place, was overturned in a carriage, and apprehended that he had dislocated his shoulder. After careful examination, my friend pronounced the stranger to be free from injury. The other coolly inquired, what he was to pay? The surgeon, somewhat piqued, answered, 'Nothing,' and walked away. It came to his knowledge, soon afterwards, that this person related the circumstance of his having for once met with *an honest surgeon*, who declined taking a fee when he found there was no work to be performed! Without referring to the study and expense the practitioner had been put to ere he could acquire the knowledge requisite to enable him to pronounce an opinion of a nature to set the fellow's mind at ease, I shall be uncharitable enough to suppose, that such a narrow-minded being would have been the very person to have brought an action for *mala praxis* had he suffered inconvenience from an erroneous opinion." 176.

At page 180, Dr. Smith enters pretty fully, but, we think, not very perspicuously, into the celebrated trial of Captain Donellan, for the murder of Sir Theodosius Boughton, concluding, apparently, that the verdict was just—contrary to the opinion of John Hunter and some other distinguished men of that and the present time.

We were rather gratified to find that the prognostication conveyed in the following passage was rather premature, since, at the moment we are writing this, we find Dr. Smith announced as a lecturer on medical jurisprudence at the Royal Institution.

"For several years I had entertained the hope that, by a sedulous cultivation of the literature, and an attentive application to the practical bearings of this science, I might qualify myself, in some measure, for aiding those whom it equally concerns to prepare for the difficulties and perplexities that ordinarily harrass the medical witness. Under this impression, I have spent not only time and labour, but other things that men sometimes value above these, in the preparation; without seeing that there is a present prospect of success—even as to an opportunity of lending a helping hand. It is a slur, perhaps, on the profession—but it is the truth—that, had I proceeded to offer such a course of Lectures as alone I would pretend to offer, the signs of encouragement were greater from the juridical than from the medical quarter of those whom such a course of instruction might concern. For the gratification of my own mind, I shall not cease to express my views of topics that I have now acquired some means of investigating; but I apprehend that my labours must be confined hereafter to books—or to parallel modes of imparting any acquisitions I may persuade myself, from time to time, would be worthy of submission to the reading portion of the medical community." 190.

We now come to Dr. Smith's laborious collection of trials—which are not only curious and interesting, as historical and judicial documents of human depravity and retributive justice—but often instructive, as exhibiting the phenomena resulting from poisons exhibited or injuries inflicted, with the view of destroying life. It cannot be supposed that we can attempt any thing like an analysis of such a mass of judicial proceedings; yet it may not be uninteresting to glance at a few of them, before we close this article.

1. The first on the list is the trial of Miss Blandy, for administering arsenic to her own father! A Scotch officer, of the name of Cranstoun, had furnished her with a powder, which, if administered to her father, was to have the effect of making him relent and give consent to their marriage! It is probable that the infatuated girl was not aware of the dreadful work she was doing. At least she declared so on the scaffold. The trial took place, about 70 years ago, at Oxford—and the chemical evidence of Dr. Addington and Dr. Lewis shews how much toxicology has improved since their time. The symptoms exhibited by the unfortunate victim of the execrable Cranstoun, were those which are now well known to follow the exhibition of arsenic, and there is every reason to believe that the wretched daughter and unnatural parricide suffered justly the death of ignominy on the gallows.

2. The trial of Elizabeth Fenning, in 1815, for mixing arsenic with dumplings, with intent to kill and murder, contains such acute commentaries on the medical evidence delivered in this case, that we are inclined to give it as it stands in the Appendix, hoping it may have a salutary influence on those who may appear in the character of witnesses in future.

" *Case of* ELIZABETH FENNING, *tried at the* Old Bailey, *April* 11*th*, 1815, *for administering Poison, with intent to Kill and Murder.**

" It is necessary to premise—that the prisoner was charged with having mixed arsenic with yeast dumplings. The first witness (her mistress) described the dough as not rising when placed before the fire for that purpose; that its shape was singular, and that this shape was retained till the last. When the dumplings had been boiled, and were brought to the table, the witness observed that they were black and heavy, and, soon after eating part of one of them, she was seized with illness; and her father and husband, who likewise partook of them, also sickened.

*Mr. Orlibar Turner* deposed, that he had been taken ill after partaking of the dumplings in question: that, entertaining suspicions of arsenic, he next morning made a search: that he added water to the remains of the dumplings in the pan in which the dough had been mixed, and that, after stirring the whole together, and setting down the pan for

" * From a pamphlet, entitled 'Important Results of an Elaborate Investigation into the Mysterious Case of Elizabeth Fenning, &c. by John Watkins, L.L.D.' In this publication, the report is stated to be much more minute than those published elsewhere.

" The editor, or author, styles the affair 'one of the most extraordinary cases that ever happened in a civilised state.'—*Preface.*"

half a minute, he discovered a white powder at the bottom of it, which he locked up till *Mr. Marshall*, the apothecary, came: that arsenic had been kept in a drawer in the office, in two wrappers tied round, and 'Arsenic, deadly poison,' written on it: that the prisoner could read and write very well: that the drawer had always remained open, and that any person might have access to it: that he had kept two of the knives with which the dumplings had been cut, and that *he had observed them black the next day*: the arsenic he subsequently stated to have been kept for the purpose of destroying mice.

"*Mr. John Marshall* sworn.—I am a surgeon. On the evening of Tuesday, the 21st of March, I was sent for to *Mr. Turner's* family: I got there about a quarter before nine: all the symptoms attending the family were produced by arsenic: *I have no doubt of it, by the symptoms*: the prisoner was also ill, by the same, I have no doubt.—Did *Mr. O. Turner* shew you a dish the next morning? He did; I examined it; I washed it with a tea-kettle of warm water; I first stirred it, and let it subside; I decanted it off; I found half a tea-spoonful of white powder; I washed it the second time; I decidedly found it to be arsenic.—Will arsenic, cut with a knife, produce the appearance of blackness upon the knife? *I have no doubt of it.**

"* The following is part of the comment on this gentleman's evidence:—'Mr. M. deposes, that there was, in the remains of the dough sticking round the dish in which the *Turners'* dumplings were made, half a tea-spoonful of arsenic. If the arsenic was mixed with the flour at the time of making the dough, it was doubtless spread and incorporated throughout the whole mass in uniform proportion. Every one, in the habit of going into the kitchen, knows about how much dough is left in a dish, after making dumplings; if collected, it would hardly exceed the size of a walnut, or one-eighth of a dumpling. If, therefore, there was in that quantity half a tea-spoonful of arsenic, which, it has been ascertained, would weigh at least fifty grains, there would have been, in the four dumplings and a half actually eaten, a quantity of arsenic weighing 1,800 grains. Now, as five grains of arsenic would destroy any human being who swallowed it, the quantity in *Mrs. Turner's* quarter of a dumpling was equal to the death of ten persons; that in her husband's dumpling and a half would have killed 120; and if *Mr. O. Turner* and *E. Fenning's* proportions of dumpling were alike, each of theirs held a proportion equal to the death of 110 persons; so that the quantity of arsenic in the four dumplings and a half, would have destroyed 360 people! The large portion of arsenic, therefore, in the small quantity of dough remaining in the dish after making the dumplings, is only to be accounted for by supposing that a portion of arsenic was sprinkled or strewed upon the surface of the dough whilst in the pan or dish before the fire; in which case, upon making the dough into dumplings, although the greater quantity would be incorporated, yet a considerable portion would fall off into the dish. * * * * * Mr. M. found it to be *arsenic*, but he has not stated how he *knew* it to be so. Did Mr. M. by mere inspection, find it to be so? Or did he find it to be so on the authority of any other person? Did he *test* it, and when? What *tests* did he use? What *became* of the arsenic? * * * Was the dish the only vessel, except the flour-tub and yeast-basin, that Mr. M. examined? Did it not occur to him to examine the pot in which the dumplings were boiled?

"According to the principle upon which this Appendix is constructed—that of avoiding, as entirely as possible, all extra-professional testimony, and moral considerations, I have confined myself closely to the medical relations of this investigation. Of Dr. Watkins I have not the least personal knowledge. At the time of the transaction, I was engaged in the busy scenes that were passing near Brussels and at Paris—inattentive to the politics of the Old Bailey, and ignorant of this particular event, any farther than a slight acquaintance with newspaper records might have prevented—but of which I retain not the slightest consciousness. I did not revisit England for more than three years afterwards, and my attention was not directed to the case until a still longer period had elapsed—so that I may safely declare myself free from all bias with regard to persons and interests. Looking at its merits as a medico-legal investigation, I cannot but feel deeply interested; and do consider it right to direct those who may wish for a full and important lesson on the nature and abuse of testimony, to consult this very curious and able pamphlet. It gives a vivid lesson on the duties of a medical witness, whatever may have been the truth as to the guilt of the party accused. I shall restrict any farther use of it, in this place, to the following extract.

"'*Effects of Arsenic upon Yeast Dough*.—That part of the evidence relative to the weight and colour of the dumplings, and particularly of

---

What became of the water they were boiled in? Was there any more arsenic held in solution in that water, after a quarter of an hour's boiling of the dumplings, than would have escaped from them? Did Mr. M. inquire where the water was got from that the dumplings were mixed with, and did he inspect the vessel it was fetched in? Did he examine the milk-can that hung in the kitchen? and the salt-vessel from which the salt was taken for the dumplings? Did he examine the sauce? * * *.'

"'*Mr. Marshall* is asked if arsenic, cut with a knife, will produce the appearance of blackness upon the knife? He immediately answers, '*I have no doubt of it.*' Was *Mr. Marshall* aware that he was giving evidence and opinions upon oath, which from him, *as a professional man*, the jury would assume as true; but which, if they had been uttered by a man not professional, would have had no weight with them? Did not *Mr. Marshall* know, when he swore he had *no doubt* arsenic would turn a knife black, that the jury would believe, upon that oath, that arsenic *would* turn a knife black?' * * * * 'Mr. Marshall swore that ALL THE SYMPTOMS attending the family WERE PRODUCED BY ARSENIC; he had no doubt of it—BY THE SYMPTOMS! He likewise swore, that the white powder in the dish he decidedly FOUND to be ARSENIC; and he also swore—that he had *no doubt* ARSENIC would BLACKEN a knife.'

"The acuteness and pertinence of these questions require no elucidation; but as they do not come from an ostensibly professional quarter, I think it my duty to refer to opinions that have been expressed by proper judges of the medical bearings of this part of the case, viz. the verification of the poison. The reader may consult the reviews of Mr. Marshall's own subsequent publications—particularly that in the London Medical Repository, vol. viii. p. 158—and the Edinburgh Medical and Surgical Journal, vol. xiii. p. 518." 209.

*Mrs. Turner's* evidence, manifestly tended to persuade the jury that their heaviness and blackness were in consequence of arsenic being in the *dough;* a persuasion, the effect of most loose and erroneous reasoning, and entirely devoid of rational support. *If* the dumplings were *poisoned* at all, and there is NO EVIDENCE that they were—*if* they were poisoned with *arsenic*, and no witness proves that there was a single grain of *arsenic* in the *dumplings;* but, admitting that they were, the reasonable presumption is, that the arsenic was not incorporated in the dough at the time of the making, but that it was sprinkled or strewed on, after the dough was put before the fire *to rise.*

"'Now, it is by no means difficult to incorporate arsenic with dough prepared for dumplings, commonly called *yeast* dumplings, *after the first mixing* of the ingredients, so as to render the dough poisonous to any person who may eat of it. The *colour* of *arsenic* is not different from the colour of *flour;* one resembles the other so closely, that none but a person acquainted with the peculiar characteristics of *arsenic* can distinguish it from flour, even when *casually* sprinkled, *still less* when the two substances are mixed together.

"'*Arsenic* mixed with dough containing *yeast*, will *not* prevent the mixture from *rising*, ALTHOUGH the quantity of *arsenic* EXCEED TWO THIRDS *of the mass.* It is generally known, that *yeast* contains a large quantity of *carbonic acid gas* in a concentrated state: the effect of heat extricates the bubbles of *gas*, and, in the act of extrication, distends the dough until all further attraction for *caloric*, or heat, ceases, by the total absence of *gas.* In this state, if the mass be confined at its sides, its surface will become elevated, and present the appearance of what is termed *rising.*

"'It is evident, that to prevent dough from *rising*, the extrication of *carbonic acid gas* by *caloric*, or heat from the fire, must also be prevented; and this can only be done by saturating the *gas* with an *alkali*, thereby breaking down the chemical *aggregation* which is produced by the affinity of an acid to an alkali. Arsenic not being an alkali, and, therefore, incapable of saturating *carbonic acid gas*, it *cannot* prevent dough, or any other matter containing *carbonic acid gas*, from *rising*, when exposed to the action of *caloric* or heat.* Hence it is clear, that so much of the recorder's charge to the jury as instructed them that the heaviness and black appearance of the dumplings were occasioned by the arsenic, was nugatory, and unsupported by fact or experience.'

"'*Effects of Arsenic upon the Knives.*—That arsenic did not blacken the *two* knives produced by *Mr. O. Turner* on the trial, out of the *three* used up stairs at dinner, is as certain as that *Mr. Marshall* swore it would blacken them. A yeast dumpling, compounded with a very large proportion of arsenic, was boiled, and afterwards cut to pieces with a knife, purposely cleaned. The knife was carefully put by, with whatever of the dumpling remained on its sides after cutting; when dry, the crumbs were removed, and there was not the least blackness on the knife.

"* A note here states these conclusions to be the *result of experiments.*"

" ' A gentleman of chemical eminence, in the city, put more arsenic into a pint of water than could be held in solution, and boiled it at a sand heat. A clean knife being placed in the water while hot, remained there until it was cold. The knife was then taken out wet, and remained untouched until the blade became perfectly dry. It was in no way whatever discoloured.

" ' Arsenic, moistened with water, has been formed into a sort of paste, and placed upon the blade of a knife to dry there, without producing any discoloration on the surface of the blade. Arsenic, moistened with water, has been rubbed upon the blade of a knife with the fingers, and suffered to dry on, without changing the colour of the steel. The production of the *two* blackened knives, therefore, was no more proof of the presence of arsenic in the dumplings, than *Mr. Marshall's* testimony to that effect.' " 211.

The celebrated trial, at Launceston, of Mr. Donnall, surgeon, for the murder of Mrs. Downing by poison, occupies 20 pages of Dr. Smith's work, and is a good exemplification of the discrepancies of medical testimony in Courts of Justice. We know not whether the Judge and Jury were enlightened or darkened by the professional statements in this case—we should suppose they were not a little bewildered. Dr. Smith must expect and wish that his book shall descend to posterity. There is one defect, then, which we occasionally see—namely the omission of the final issue of the trial. In Mr. Donnall's case, for instance, we are left in the dark whether he was hanged or acquitted. The trial, it is true, is too recent not to be remembered—but will it be fresh in memory 30 or 40 years hence?

Our limits are already over-stepped, and we must bring this article to a termination. From page 250 to page 267 is given the far-famed trial of Captain Donellan for the exhibition of laurel water to Sir Theodosius Boughton. We have read the medical testimony with attention, and must say that we entertain little or no doubt that poison was administered and caused the Baronet's death. We are also constrained to say that the evidence of Mr. John Hunter, on this occasion, was a disgrace to such a man. But we will give it here, together with Dr. Smith's commentary, as a curious piece of forensic history.

" *Mr. John Hunter* sworn.* 1. Have you heard the evidence that has been given by these gentlemen? I have been present the whole time.—2. Did you hear *Lady Boughton's* evidence? I heard the

" * The questions put to Mr. Hunter are numbered, for the reason assigned in the former part of the Appendix, (page 187,) and for the more ready application of the subjoined remarks, which I have thereby been enabled to abbreviate materially."

whole.—3. Did you attend to the symptoms her ladyship described as appearing upon *Sir Theodosius Boughton*, after the medicine was given him? I did.—4. Can any certain inference, upon physical or chirurgical principles, be drawn from those symptoms, or from the appearances, externally or internally, of the body, to enable you, in your judgment, to decide that the death was occasioned by poison? I was in London then; and a gentleman who is in court waited upon me with a copy of the examination of *Mr. Powell* and *Lady Boughton*, and an account of the dissection, and physical gentlemen's opinion upon that dissection.—5. I don't wish to go into that; I put my question in a general way? The whole appearances upon the dissection explain nothing but putrefaction.—6. You have long been in the habit of dissecting human subjects? I presume you have dissected more than any man in Europe? I have dissected some thousands during these thirty-three years.—7. Are those appearances you have heard described, such, in your judgment, as are the result of putrefaction in dead subjects? Entirely.—8. Are the symptoms that appeared after the medicine was given, such as necessarily conclude that the person had taken poison? Certainly not.—9. If an apoplexy had come on, would not the symptoms have been nearly or somewhat similar? Very much the same.—10. Have you ever known or heard of a young subject dying of an apoplectic or epileptic fit? Certainly; but, with regard to the apoplexy, not so frequent; young subjects will perhaps die more frequently of epilepsies than old ones; children are dying every day from teething, which is a species of epilepsy arising from an irritation.—11. Did you ever, in your practice, know an instance of laurel-water being given to a human subject? *No, never.*—12. Is any certain analogy to be drawn from the effects of any given species of poison upon an animal of the brute creation, to that it may have upon a human subject? As far as my experience goes, which is not a very confined one, because I have poisoned some thousands of animals, they are very nearly the same; opium, for instance, will poison a dog similar to a man, arsenic will have very near the same effect upon a dog, as it would have, I take for granted,* upon a man; I know something of the effects of them, and I believe their operations will be nearly similar.—13. Are there not many things which will kill animals almost instantaneously, that will have no detrimental or noxious effect upon a human subject; spirits, for instance, occur to me? I apprehend a great deal depends upon the mode of experiment; no man is fit to make one, but those who have made many, and paid considerable attention to all the circumstances† that relate to experiments. It is a common experiment, which I believe seldom fails, and it is in the mouth of every body, that a little brandy will kill a cat: I have made the experiment, have killed several cats, but it is a false experiment; in all those cases where it kills the cat, it

"* Loose language enough, as *testimony*."

"† This observation is founded in good sense; it bears out the advice given in the text, page 140."

kills the cat by getting into her lungs, not into her stomach; because if you convey the same quantity of brandy, or three times as much, into the stomach, in such a way as the lungs shall not be affected, the cat will not die; now, in those experiments that are made in forcing an animal to drink, there are two operations going on, one is a refusing the liquor by the animal, its kicking and working with its throat to refuse it; the other is the forcing the liquor upon the animal; and there are very few operations of that kind, but some of the liquor gets into the lungs; I have known it from experience.—14. If you had been called upon to dissect a body suspected to have died of poison, should you or not have thought it necessary to have pursued your search through the guts? Certainly.—15. Do you not apprehend that you would have been more likely to receive information from thence than any other part of the human frame? That is the track of the poison, and I should certainly have followed that track through.—16. You have heard of the froth issuing from *Sir Theodosius*'s mouth a minute or two before he died; is that peculiar to a man dying of poison, or is it not very common in many other complaints? I fancy it is a general effect of people dying in what you may call health, in an apoplexy, or epilepsy; in all sudden deaths, where the person was, a moment before that, in perfect health.—17. Have you ever had an opportunity of seeing such appearances upon such subjects? Hundreds of times.—18. Should you consider yourself bound, by such an appearance, to impute the death of the subject to poison? No, certainly not; I should rather suspect an apoplexy, and I wish, in this case, the head had been opened to remove all doubts.—19. If the head had been opened, do you apprehend all doubts would have been removed? It would have been still farther removed, because, although the body was putrid, so that one could not tell whether it was a recent inflammation, yet an apoplexy arises from an extravasation of blood in the brain, which would have laid in a coagulum. I apprehend, although the body was putrid, that would have been much more visible than the effect any poison could have had upon the stomach or intestines.—20. Then, in your judgment, upon the appearances the gentlemen have described, no inference can be drawn from thence that *Sir Theodosius Boughton* died of poison? Certainly not, it does not give the least suspicion.

"(Cross-examined by *Mr. Howorth.*) 21. Having heard the account to-day, that *Sir Theodosius Boughton*, apparently in perfect health, had swallowed a draught which had produced the symptoms described, I ask you, whether any reasonable man can entertain a doubt that that draught, whatever it was, produced those appearances? I don't know well what answer to make to that question.—22. Having heard the account given of the health of this young gentleman on that morning, previous to taking the draught, and the symptoms that were produced immediately upon taking the draught, I ask your opinion, as a man of judgment, whether you don't think that draught was the occasion of his death? With regard to his being in health, that explains nothing; we frequently, and indeed generally, see the healthiest people dying suddenly, therefore I shall lay little stress upon that: as to the circumstances of the draught, I own they are suspicious; every man is as good a judge as I am.—*Court.* You are to give your opinion upon the symptoms only, not upon any other evidence given.—23. *Mr. Howorth.* Upon the symptoms immediately produced, after the swallowing of that draught, I ask whether, in your judgment and opinion, that draught did not occasion his death? I can only say, that it is a circumstance in favour of such an opinion.—24. *Court.* That the draught was the occasion of his death? No; because the symptoms afterwards are those of a man dying, who was before in perfect health; a man dying of an epilepsy or apoplexy, the symptoms would give me those general ideas.—25. It is the general idea you are asked about now; from the symptoms which appeared upon *Sir Theodosius Boughton* immediately after he took the draught, followed by his death so very soon after; whether, upon that part of the case, you are of opinion that the draught was the occasion of his death*? If I knew the draught was poison, I should say, most probably, that the symptoms arose from that; but when I don't know that that draught was poison, when I consider that a number of other things might occasion his death, I cannot answer positively to it.—26. You recollect the circumstance that was mentioned of a violent heaving in the stomach? All that is the effect of the voluntary action being lost, and nothing going on but the involuntary. —27. *Mr. Howorth.* Then you decline giving any opinion upon the subject? I don't form any opinion to myself; I cannot form an opinion; I can conceive, if he had taken a draught of poison, it arose from that; I can conceive it might arise from other causes.—28. If you are at all acquainted with the effects and operation of distilled laurel-water, whether the having swallowed a draught of that, would not have produced the symptoms described? I should suppose it would; I can only say this, of the experiments I have made of laurel-water upon animals, it has not been near so quick; I have injected laurel-water directly into the blood of dogs, and they have not died; I have thrown laurel-water, with a precaution, into the stomach, and it never produced so quick an effect with me as described by those gentlemen.—29. But you admit that laurel-water would have produced symptoms such as have been described? I can conceive it might.—30. *Mr. Newnham.* Would not an apoplexy or an epilepsy, if it had seized *Sir Theodosius Boughton* at this time, though he had taken no physic at all, have produced similar symptoms too? Certainly.—31. Where a father has died of an apoplexy, is not that understood, in some measure, to be constitutional? There is no disease whatever that becomes constitutional, but what can be given to a child. There is no disease which is acquired,

"* It is almost incomprehensible, how a man of any acuteness, or even one without acuteness, who should pay but ordinary attention to such a jumbled question, would proceed to answer it. Here a lawyer sets out with a declaration that he asks a question about 'the general idea,' (which in itself carries no meaning,) and then specifies *a particular fact* on which the question is really built."

that can be given to a child; but whatever is constitutional in the father, the father has a power of giving that to the children; by which means it becomes what is called hereditary: there is no such thing as an hereditary disease, but there is an hereditary disposition for a disease*. 32. *Mr. Howorth.* Do you call apoplexy constitutional? We see most diseases are constitutional; the small-pox is constitutional, though it requires an immediate cause to produce the effects. The venereal disease is hereditary. I conceive apoplexy as much constitutional as any disease whatever.—33. Is apoplexy likely to attack a thin young man who had been in a course of taking cooling medicines before? Not so likely, surely, as another man; but I have, in my account of dissections, two young women dying of apoplexies.—34. But in such an habit of body, particularly attended with the circumstance of having taken cooling medicines, it was very unlikely to happen? I do not know the nature of medicines so well as to know that it would hinder an apoplexy from taking an effect.—35. *Court.* Give me your opinion, in the best manner you can, one way or the other, whether, upon the whole of the symptoms described, the death proceeded from that medicine, or any other cause? I do not mean to equivocate, but when I tell the sentiments of my own mind, what I feel at the time, I can give nothing decisive.'

"I shall now add the remaining part of the commentary which I have ventured to insert upon this important passage in medico-legal history, and which relates entirely to the course of examination pursued with Mr. Hunter.

"Had he been allowed to proceed with the intended answer 4, something would perhaps have come out that would have given a different turn to the whole affair. A simple query from *Mr. Howorth,* as to the specific use *Mr. Hunter* had made of the information communicated at that time, would have brought out something probably worth knowing. The answer to 10 is nothing to the purpose. Apoplexy is not the disease of young men nearly twenty-one†; and the nature of epilepsy does not favour the supposition of a person going off in *the first* fit of that disease at such a period of life. Answer 13 is mere bye-play; and though 14 may appear very pertinent, such an examination, however desirable to have been made, when the issue undergoes a mystification on account of its neglect, is not universally necessary, particularly in cases of vegetable poisoning, such as that in question. The answer to 18 is specious enough; but if the head had been opened, and congestion had been found, would Mr. Hunter have maintained that such an appearance discountenanced the supposition of death by narcotic poison? As to coagula (19), the statement that extravasation *must* take place in apoplexy, was (to say the least of it) loose on the part of such an authority. The answer to 22 is decidedly favourable to the evidence on behalf of the crown. Contrast it with that of 20—it savours of contradiction. With regard to questions 23, put by the counsel for the crown, and 24,

"* See Principles, page 521." "† Look at the answer 33."

put (for the more certain understanding of the other) by the judge, is there not a flat contradiction in the answers, followed by an unintelligibly constructed sentence in place of an explanation? At 24 he seems inclined to evade, by an incomprehensible allusion to the symptoms of persons dying in perfect health; as if there were established symptoms of that nature: if the words are correctly taken down, what do they mean? Have they any meaning at all? At 25 he says, '*If* I knew'—here he must mean of his own knowledge, from having made the draught up himself, for he discredits the crown witnesses if he did *not* know. As to 26, I ask whether narcotic poisons do *not* suppress voluntary action? What does he say to 27? He extinguishes his own torch completely. 28 is merely waiving the question, and to 29 he says fully as much against the prisoner as any thing uttered by him in the course of the examination, or his whole evidence taken together, could possibly be construed to make in his favour. And when at last urged for an opinion of any sort, either way, (35,) what does it all come to? 'I can give nothing decisive.'

"Now, whether the unfortunate prisoner suffered wrongfully or not, I will not take upon me to infer; but I cannot help thinking, that the evidence of the only professional witness brought forward on his behalf, should go for very little, and that, as the ground for forming an opinion, it is not at all to be entertained, when contrasted with that adduced on the part of the prosecution." 267.

We recommend the work, more especially the Appendix, to our brethren, as containing a CORPUS HISTORICUM of medical evidence, remarkably well calculated to excite to the study of forensic medicine, in its various and extensive bearings. The profession is under a great obligation to Dr. Smith for the labour which he has bestowed upon the work in general, and the Appendix in particular.

